# Supplementary material for: Porphyromonas gingivalis outer membrane vesicles alter cortical neurons and Tau phosphorylation in the embryonic mouse brain
Source: PLoS One. 2025 Mar 11;20(3):e0310482. doi: 10.1371/journal.pone.0310482 (PMC11896034; doi:10.1371/journal.pone.0310482)
Supplement: S2 File — (PDF) [file pone.0310482.s002.pdf]

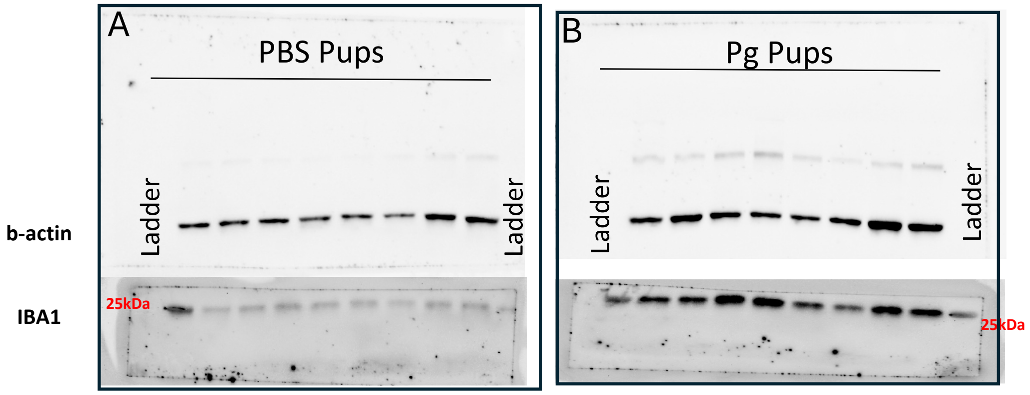

Figure S2 A and B: Iba-1 and β-actin western blots.

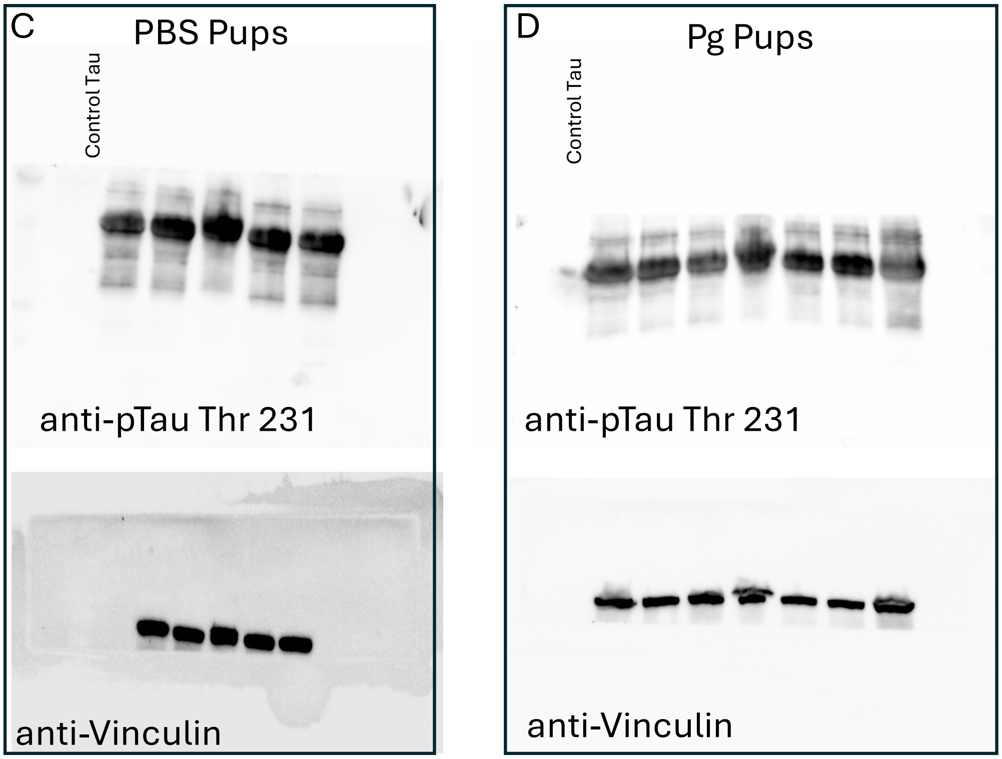

Figure S2 C and D: anti-pTau Thr231 and vinculin western blots. The control Tau protein is not phosphorylated at Thr231.

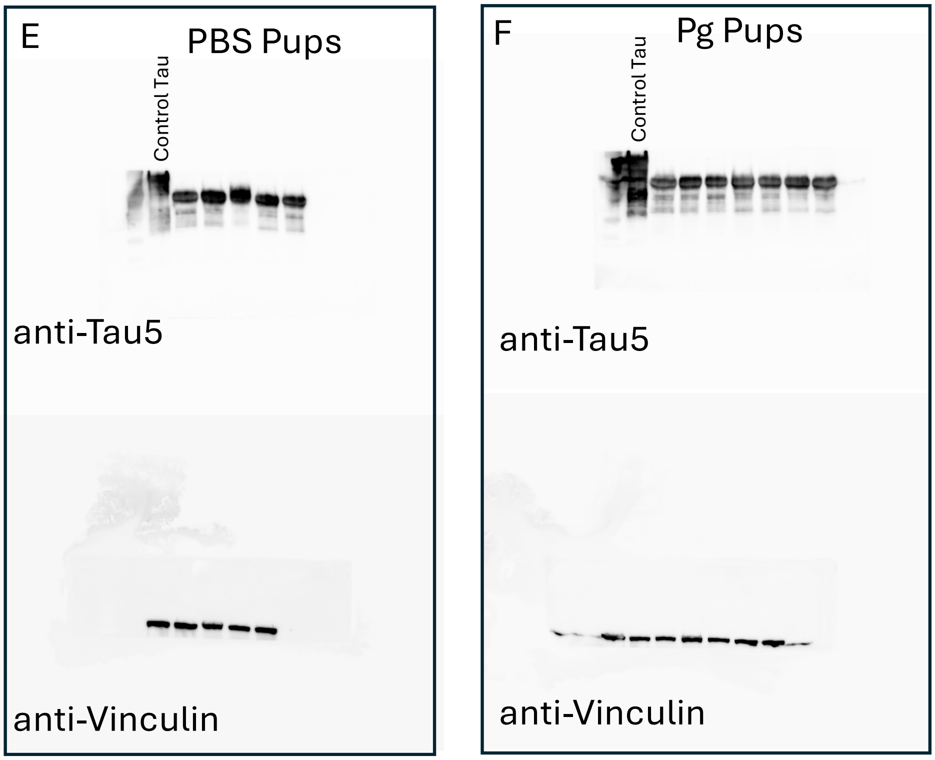

Figure S2 E and F: anti-Tau5 and vinculin western blots.
