# Supplementary material for: Porphyromonas gingivalis outer membrane vesicles alter cortical neurons and Tau phosphorylation in the embryonic mouse brain
Source: PLoS One. 2025 Mar 11;20(3):e0310482. doi: 10.1371/journal.pone.0310482 (PMC11896034; doi:10.1371/journal.pone.0310482)
Supplement: S1 File — (PDF) [file pone.0310482.s001.pdf]

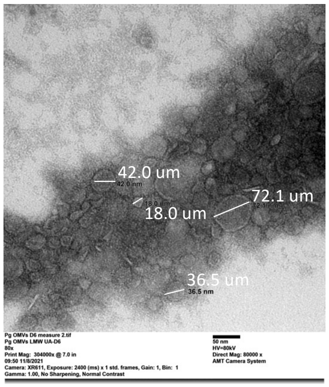

Supplemental Figure 1A. Representative TEM Image of Pg-OMVs. Lines and values for particle diameter are enhanced for better visibility.

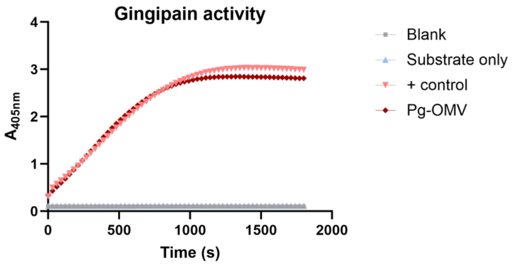

Supplemental Figure 1B. Time course of gingipain assay for OMV prep. L-BAPNA was used as a substrate with live Pg as positive control using the method of Potempa and Nguyen, Current Protocol on Protein Science, 2007 . The blank and substrate only values overlap on the y-axis.

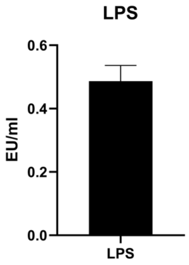

Supplemental Figure 1C. LPS quantification. LPS was quantified by the Pierce™ Chromogenic Endotoxin Quant Kit According to the manufacturer’s instructions. LPS concentration was 0.49 ± 0.09 EU/ml (endotoxin units/ml). Roughly 1 EU is 1-2 ng of LPS. LPS
